# The Neutrophil–Lymphocyte Ratio Is Associated with Cardiac Magnetic Resonance Imaging-Derived Myocardial Fibrosis

**DOI:** 10.3390/jcm15041441

**Published:** 2026-02-12

**Authors:** Michael Poledniczek, Christina Kronberger, Lena Marie Schmid, Katharina Mascherbauer, Carolina Donà, Matthias Koschutnik, Laura Lunzer, Christian Nitsche, Dietrich Beitzke, Christian Loewe, Christian Hengstenberg, Andreas Anselm Kammerlander

**Affiliations:** 1 Division of Cardiology, Department of Internal Medicine II, Medical University of Vienna, 1090 Vienna, Austria; michael.poledniczek@meduniwien.ac.at (M.P.);; 2 Division of Cardiovascular and Interventional Radiology, Department of Biomedical Imaging and Image-Guided Therapy, Medical University of Vienna, 1090 Vienna, Austria

**Keywords:** neutrophil–lymphocyte ratio, cardiovascular risk, inflammation, biomarkers, myocardial fibrosis, cardiac magnetic resonance imaging

## Abstract

**Background**: Sub-clinical inflammation is considered a key mechanism in cardiovascular disease and myocardial remodeling. We therefore evaluated whether the neutrophil–lymphocyte ratio (NLR), a simple inflammatory marker derived from a routine full blood count, is associated with myocardial fibrosis. **Methods**: Consecutive patients from a cardiac magnetic resonance imaging (CMR) registry were included and stratified by the NLR tertile. The association of the NLR and the levels of C-reactive protein (CRP) with CMR-derived myocardial T1 time and the extracellular volume fraction (ECV) were assessed using linear regression analysis and compared using Z-scores. In addition, an association with outcome was tested utilizing the log-rank test. **Results**: 1152 patients (72.4 years, 53.1% male) constituted the final cohort. The median NLR was 3.11 [interquartile range (IQR): 2.145–4.67]. Tertiles were based on the cut-off values ≤ 2.5, >2.5 and <4.0, and ≥4.0. A higher NLR tertile was associated with lower biventricular ejection fraction, hypertrophy, and increased right ventricular volume. The myocardial native T1 time [tertile 1 vs. 3: 1010 ms (984–1038) vs. 1030 (1001–1059), *p* < 0.001] and ECV [tertile 1 vs. 3: 26.3% (24.2–28.6) vs. 28.1% (25.5–31.4), *p* < 0.001] also significantly differed between the NLR tertiles. The NLR’s and the CRP’s association with elevated myocardial T1 time and ECV were comparable; however, the proportion of variation in the target variables explained by either was generally low. **Conclusions**: In our CMR all-comer cohort, NLR and CRP were significantly associated with prolonged myocardial T1 times and increased ECV. However, only a modest variation observed in both parameters was explained by either variable.

## 1. Introduction

Sub-clinical inflammation is considered a crucial aspect, as well as a propagator of various diseases, including cancer, diabetes mellitus, and atherosclerosis [1,2]. Sterile, low-grade inflammation is tightly linked to aging, cellular loss of function, and replacement fibrosis [3]. In the context of cardiovascular disease, pro-inflammatory mediators promote arterial stiffness, myocardial remodeling, and atherosclerosis [3].

Development of myocardial fibrosis has been suggested as another detrimental effect of chronic inflammation and may be observed in hypertrophic or dilated cardiomyopathy or following myocardial infarction [4,5,6,7]. In patients with heart failure, chronic inflammation was previously linked to disease progression and poor outcome independent of conventional markers of disease severity, including the New York Heart Association functional class and left ventricular ejection fraction [4]. In addition to mechanical impairment of myocardial compliance and contractility, myocardial fibrosis also promotes both atrial and ventricular arrhythmia [8]. Furthermore, interstitial fibrosis results in impaired oxygen supply due to increased diffusion distances [8]. As myocardial fibrosis can be observed in various diseases, estimation of the true prevalence of this finding remains challenging. This is further complicated by the fact that no cut-offs for defining myocardial fibrosis currently exist.

While attenuation of inflammation would be an attractive therapeutic target in these patients, to date, clinical trials of anti-inflammatory agents have demonstrated mixed results [4,9,10]. Colchicine, which affects tubulin polymerization and responsiveness of leukocytes, has demonstrated the ability to reduce cardiovascular events in patients with chronic coronary artery disease [10]. On the other hand, canakinumab, a selective inhibitor of interleukin 1β, reduced recurrent cardiovascular events in patients who experienced myocardial infarction and demonstrated elevated levels of C-reactive protein (CRP) in serum [9]. However, this came at the cost of an increased rate of fatal infections and, overall, no effect on all-cause mortality [9].

It may be hypothesized that trials on inflammation in cardiovascular disease currently lack appropriate biomarkers that can be utilized to select those patients who are most suitable to benefit from such therapies. The neutrophil–lymphocyte ratio (NLR) is a simple and easily assessable biomarker of inflammation and has been suggested to be independently associated with outcome in patients with heart failure beyond conventional markers of inflammation [11,12], as well as in patients receiving transaortic valve replacement interventions [13].

Cardiac magnetic resonance imaging is the gold standard of non-invasive diagnostic imaging modalities used to accurately characterize the myocardium [14]. In cardiac magnetic resonance imaging (CMR), fibrosis and myocardial remodeling can be assessed utilizing T1 parametric mapping techniques [14]. In addition, with the support of gadolinium-based contrast agents, which accumulate in the extracellular space, the post-contrast T1 time can be utilized to calculate the extracellular volume fraction (ECV) [14]. CMR-based ECV has been validated against histological assessment [15] and may be used to detect myocardial fibrosis more accurately than T1 mapping alone [14].

We therefore aimed to investigate the potential link between the NLR as a simple and affordable biomarker of sub-clinical inflammation and CMR-based assessment of myocardial fibrosis. We furthermore compare the supposed association of the NLR with that of CRP. Finally, we assessed the association of the target parameters with all-cause mortality.

## 2. Materials and Methods

### 2.1. Settings and Subjects

This post hoc analysis was conducted within the scope of a prospective CMR registry established at the Medical University of Vienna, Department of Internal Medicine II, Division of Cardiology. The registry was approved by the local institutional review board (decision no. 2036/2015) and was implemented in accordance with the principles outlined in the Declaration of Helsinki. All patients provided evidence of informed consent per personal signature.

Adult patients included in the CMR registry between July 2012 and August 2023 were screened for eligibility and included in the present analysis if (1) gadolinium-based contrast-enhanced imaging and parametric mapping was performed and (2) if a complete hemogram was available. Patients with (1) active infection defined as a CRP level exceeding 10 times the upper limit of normal (>5 mg/dL) or undergoing treatment for infection within the last 14 days, (2) cardiac amyloidosis or suspicion thereof, and (3) active pregnancy were excluded from the analysis.

### 2.2. Parameters of Interest

The NLR was calculated by dividing the absolute number of neutrophils (G/L) by the lymphocyte count (G/L) from the complete hemogram. In addition, conventional markers of systemic inflammation, including CRP and the absolute number of leukocytes were calculated. All laboratory analyses were performed at the central laboratory of the Department of Laboratory Medicine at the Medical University of Vienna, Vienna, Austria. Hemograms were generated with Sysmex XE and XN series hematology systems (Sysmex Corporation, Kobe, Japan). For calculation of the estimated glomerular filtration rate (eGFR), the Chronic Kidney Disease Epidemiology Collaboration equation was utilized. For all laboratory analyses, which included a complete hemogram, blood chemistry, and measurement of cardiac enzymes, including N-terminal prohormone of B-type natriuretic peptide (NT-proBNP), blood was drawn prior to the examination via a needle routinely inserted for the application of contrast agent.

Prior to CMR, routine demographic parameters, including age, weight, height, and comorbidities, were evaluated as previously described [16]. Patient history was compiled and included hypertension (defined as a non-invasively measured blood pressure in excess of 140/90 mmHg or active treatment with anti-hypertensive medication), current atrial fibrillation and history thereof, diabetes (with fasting serum glucose levels of >126 mg/dL, or a glycated hemoglobin A proportion of ≥6.5%, or pharmacologic treatment for diabetes), dyslipidemia (defined as serum cholesterol > 240 mg/dL or medication lowering cholesterol). Chronic kidney disease as was defined as an eGFR of <60 mL/min/1.73 m^2^). In addition, patient documentation was screened for diagnoses of chronic obstructive pulmonary disease, peripheral artery disease, and myocardial infarction, which was correlated with CMR findings indicative of post-ischemic scarring.

Quantitative volumetric assessments were acquired using the semi-automated Medis Suite MR (Medis Medical Imaging, Leiden, The Netherlands) operated by expert radiology technicians with more than 3 years of experience in operating CMR. Myocardial T1 relaxation times were measured in several regions of the myocardium and averaged. The interaction of myocardial T1 relaxation times with markers of chronic systemic sub-clinical inflammation were assessed. By utilizing pre- and post-contrast T1 maps, the ECV was calculated as previously described [16].

The primary outcome endpoint of this analysis was all-cause mortality, which was assessed by performing regular queries on the national statistics authority’s (Statistics Austria) death registry and by utilizing in-hospital and out-of-hospital electronic patient documentation.

### 2.3. Cardiac Magnetic Resonance Imaging

All patients referred to CMR were examined using a 1.5 Tesla scanner (Avanto FIT, Siemens Healthineers, Forchheim, Germany), adhering to standard protocols featuring gadolinium-contrast enhanced imaging, as previously described [16]. In short, inline, pixel-based T1 maps were acquired by electrocardiographically triggered modified Look-Locker inversion recovery using Myomaps^®^ (Siemens Healthineers, Forchheim, Germany). Over several heartbeats, images with shifted T1 time, inline motion correction, and inline calculation of the T1 relaxation curves within one breath hold were acquired. The following T1 sequence parameters were utilized: a starting inversion time of 120 ms, inversion time increment of 80 ms, a reconstructed matrix size of 256 × 218, and a measured matrix size of 256 × 144 (with a phase-encoding resolution of 66% and a phase-encoding field of view of 85%). T1 maps were created before and 15 min after the application of gadolinium-based contrast agent. Utilizing pre- and post-contrast T1 maps, ECV was calculated as previously described [17].

### 2.4. Statistical Analysis

All categorial variables are provided as counts and percentages. Continuous variables are presented either as mean and standard deviation (SD) or median and interquartile range (IQR), subject to the respective variables’ distributions, which were assessed using the Shapiro–Wilk test.

Patients were stratified by the NLR tertiles and compared using the chi-square test, the Kruskal–Wallis test, or one-way analysis of variance, depending on the respective variables’ properties and distributions. The association of markers of sub-clinical systemic inflammation with parameters indicative of myocardial remodeling was tested by utilizing multivariable linear regression analysis and compared by utilizing Z-scores. Z-scores were calculated by subtraction of the mean and division by the SD. The association of the target parameters with the primary endpoint was tested using the log-rank test and visualized using the Kaplan–Meier method.

Statistical significance was assumed with *p*-values of <0.05 and confidence intervals (CI) of 95%. All statistical analyses were performed using BlueSky Statistics 10.3.4, R package version 8.95 (BlueSky Statistics LLC., Chicago, IL, USA).

## 3. Results

**Figure 1:**
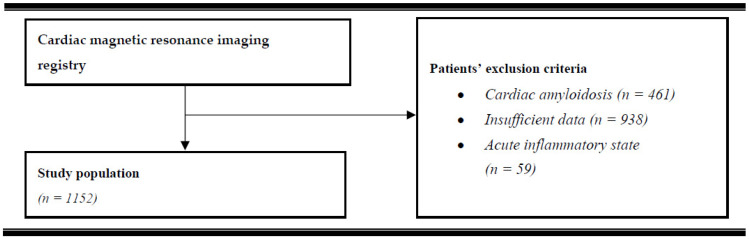
Flow chart of the patient recruitment process.

A total of 1152 patients (72.4 years of age, 53.1% male) met all inclusion and none of the exclusion criteria and formed the final study cohort. The patient recruitment process is depicted in Figure 1. When stratified into NLR tertiles, 396 patients were included in tertile 1 (NLR ≤ 2.5), 372 patients made up tertile 2 (NLR > 2.5 and <4.0), and tertile 3 comprised 384 patients with a NLR ≥ 4.0.

**Figure 2:**
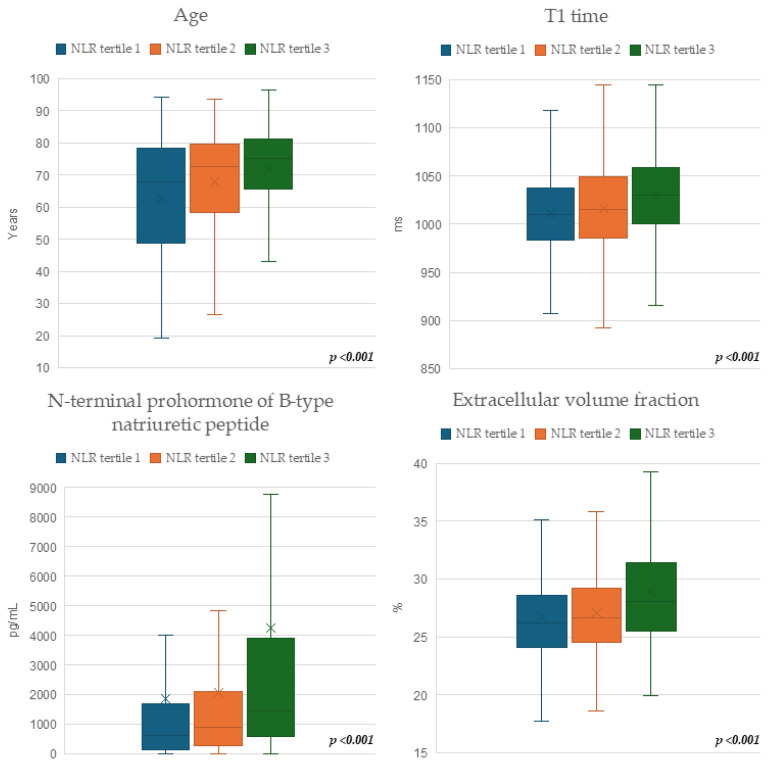
Boxplots of patients’ baseline characteristics stratified for neutrophil–lymphocyte ratio tertile. X indicates the mean.

The patient cohort’s baseline characteristics stratified by NLR tertile are depicted in Table 1 and Figure 2. Patients with a higher NLR tended to be older [tertile 1 vs. 3: 67.8 years (IQR: 48.9–78.2) vs. 75.3 (IQR: 65.7–81.2), *p* ≤ 0.001], and they more frequently had a history of atrial fibrillation, chronic obstructive pulmonary disease, diabetes mellitus, and chronic kidney disease. In addition, patients with a higher NLR were also more likely to have suffered from previous myocardial infarction (25.0% vs. 33.5% vs. 38.3%, *p* < 0.001) and to have been prescribed diuretics.

In the laboratory assessment, the median levels of CRP were significantly associated with the NLR [tertile 1 vs. 3: 0.19 mg/dL (0.08–0.43) vs. 0.41 (0.16–1.25), *p* < 0.001). Furthermore, biomarkers of cardiac, as well as renal function, were elevated in NLR tertiles 2 and 3 compared to patients in tertile 1. Table 2 shows the full patient cohort’s CMR characteristics stratified for NLR tertile. In CMR, a higher NLR tertile was associated with lower biventricular ejection fraction, hypertrophy, and increased right ventricular volume. While numerically small, a significant difference was also observed with regard to the left atrial volume indexed to body surface area [tertile 1 vs. 3: 19.6 mL/m^2^ (IQR: 17.2–22.7) vs. 20.4 (17.8–23.1), *p* = 0.027]. Importantly, the myocardial tissue native T1 time [tertile 1 vs. 3: 1010 ms (984–1038) vs. 1030 (1001–1059), *p* < 0.001] and ECV [tertile 1 vs. 3: 26.3% (24.2–28.6) vs. 28.1% (25.5–31.4), *p* < 0.001] were associated with the NLR.

In a univariable linear regression model, the NLR, age, body-mass index, history of atrial fibrillation, hyperlipidemia, and chronic kidney disease, as well as levels of CRP, NT-proBNP, and the eGFR, were significantly associated with the myocardial T1 time. In a direct comparison between the NLR’s and the CRP’s association with elevated myocardial T1 time and ECV utilizing Z-scores, the NLR Z-scores’ β were 6.38 [95%-CI: 3.58–9.17, *p* < 0.001, R = 0.017] and 0.782 (95%-CI: 0.471–1.09, *p* < 0.001, R = 0.023), respectively, whereas the CRP’s Z-scores’ β were estimated at 6.39 (95%-CI: 3.57–9.21, *p* < 0.001, R = 0.017) and 0.862 (95%-CI: 0.536–1.19, *p* < 0.001, R = 0.026). All results from the linear regression analyses for T1 time and ECV are shown in Table 3 and Table 4.

**Figure 3:**
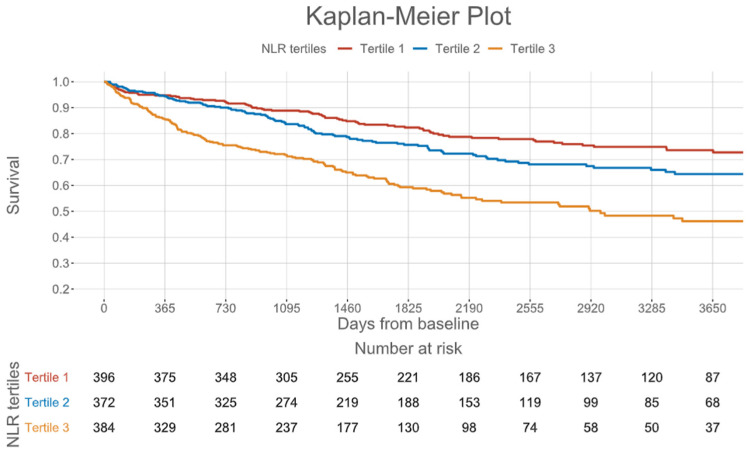
Kaplan–Meier curves by neutrophil–lymphocyte ratio tertiles for all-cause mortality; NLR indicates neutrophil–lymphocyte ratio.

When stratified in tertiles, the NLR was a highly significant indicator of outcome in this all-comer CMR cohort (log-rank test *p* < 0.001), and the corresponding Kaplan–Meier plot is depicted in Figure 3.

## 4. Discussion

In this post hoc single-center analysis, a potential association between the NLR as a biomarker of inflammation and CMR-based measurements indicative of myocardial fibrosis was tested. We could demonstrate that in our sample of CMR all-comers, a higher NLR was significantly associated with myocardial fibrosis assessed using T1 time and ECV.

While a link between biomarkers of inflammation and myocardial fibrosis has been established previously [18], this is the first analysis linking myocardial fibrosis to sub-clinical inflammation assessed using the NLR. In addition, our results also suggest a similar association between myocardial fibrosis and CRP.

The NLR has been reported as a potential prognostic marker in various cardiovascular disease entities, including valvular heart disease [13] and heart failure [11,12]. In the latter, a higher NLR has also been associated with advanced age, more severely impaired functional status, occurrence of atrial fibrillation, levels of NT-proBNP, and systolic dysfunction [11]. In heart failure with preserved ejection fraction, where inflammation and myocardial fibrosis are commonly considered hallmarks of disease [19], the NLR has been demonstrated to be independently associated with all-cause mortality, as well as a combined endpoint of all-cause mortality and heart failure-related hospitalizations [12]. In this disease, microvascular inflammation driven by pro-inflammatory metabolic comorbidities, i.e., diabetes mellitus and obesity, is suspected to result in impaired myocardial relaxation and, ultimately, heart failure [19].

Interestingly, the pathways regulating the metabolism and inflammatory responses seem to be intertwined with tumor necrosis factor, Toll-like receptor-associated and insulin mediated signaling [20]. Metabolic syndrome, which is defined as a combination of predominantly abdominal obesity, insulin resistance, diabetes mellitus, and dyslipidemia, is also associated with increased levels of biomarkers of inflammation [21]. Chronic inflammation has been demonstrated to promote the degradation of the endothelial surface layer, which consists of the endothelial glycocalyx and adsorbed plasma proteins [22]. Subsequently, the endothelial barrier function is disturbed, which promotes diapedesis of leukocytes and is suggested to be involved in the pathogenesis of various cardiovascular diseases, including atherosclerosis [23].

Contradictory to our findings, in a previous large-scale analysis, CRP has failed to demonstrate a significant association with myocardial T1 time as a surrogate for myocardial fibrosis [18]. This may be due to differences in inclusion and exclusion criteria, where patients were excluded if they reported recent conditions that could alter levels of biomarkers of inflammation. In our cohort, we applied a CRP threshold of 5 mg/dL to exclude patients with current infection or more pronounced states of systemic inflammation. This is likely to explain the marked association of CRP with myocardial fibrosis and expansion of the ECV that was suggested in our analysis. Notably, the strength of association between myocardial fibrosis and the NLR and CRP, respectively, seems rather comparable in our analysis. Interestingly, however, while an elevation in NLR is significantly associated with an elevation in CRP, the correlation between these parameters seems rather low [12]. Therefore, it may be hypothesized that the NLR and CRP are representative of different pathophysiological states and processes in the context of inflammation.

Unfortunately, there is currently no single broadly available biomarker for accurately assessing the cardiovascular risk posed by sub-clinical inflammation. CRP has been suggested to fill this gap; however, a recent large-scale meta-analysis did not confirm such an association [24]. In coronary artery disease, CRP was suggested to indicate increased risk but confer little additional value over conventional risk factors [25].

Beyond traditional CRP, high-sensitivity CRP has been shown to correlate with the overall cardiovascular risk in a healthy population, with comparable magnitude of association as non-high-density lipoprotein cholesterol or high blood pressure [26,27]. In the context of primary prevention, prescription of rosuvastatin for patients with elevated high-sensitivity CRP resulted in a reduction in relative risk of almost 50% despite only moderate baseline low-density lipoprotein (LDL) levels [26,27]. In patients who have already experienced a cardiovascular event, high-sensitivity CRP was also associated with increased risk, even in patients who achieved very low levels of LDL [26,28,29].

Therefore, lowering systemic inflammation to ameliorate its detrimental effects would be a promising concept. Indeed, in patients with previous myocardial infarction, the addition of low-dose colchicine reduced the number of cardiovascular events significantly [25,30]. In the second trial, coronary artery disease patients on 0.5 mg of colchicine experienced 31% fewer cardiovascular events [10,25], highlighting the potential of anti-inflammatory therapy in patients with coronary artery disease. However, not all trials exploring anti-inflammatory agents in the context of coronary artery disease proved successful; in a trial related to canakinumab in patients with previous myocardial infarction and levels of CRP in excess of 2 mg/dL, the interleukin 1β antagonist reduced cardiovascular events, but not all-cause mortality, due to an excess in fatal infections [9]. Despite their limited clinical impact, from the perspective of vascular biology, this study has confirmed the inflammatory nature of atherosclerosis while also promoting further research in potential anti-inflammatory therapeutics.

In the context of myocardial fibrosis and remodeling, however, prospective clinical trials regarding anti-inflammatory agents are currently lacking. Still, in animal models, sodium–glucose cotransporter 2 inhibitors have been suggested to attenuate pro-inflammatory pathways involved in myocardial apoptosis, fibrosis, and inflammation caused by treatment with doxorubicin [31]. In clinical studies, beneficial effects of sodium–glucose cotransporter 2 inhibitors in patients with heart failure with preserved ejection fraction are well established [32,33]. The exact mechanisms behind these beneficial effects remain to be fully elucidated but are suggested to be linked to the attenuation of myocardial inflammation and release of reactive oxygen species [34].

### Strengths and Limitations

As a retrospective analysis, certain limitations inherent to such studies need to be considered. First, as a tertiary referral center, the patient cohort of CMR all-comers might not be entirely representative of patient populations in other clinical settings, as referral bias could have influenced the composition of our cohort. Importantly, the effects of an elevation of the NLR and CRP, respectively, seem relatively small with an explained variation in the target parameters of <3%. Further studies are needed to find more sensitive and more specific biomarkers of systemic inflammation, which also correlate with myocardial remodeling and fibrosis. In addition, concomitant medication could not be analyzed in the present trial due to partially missing information. Finally, while smoking status or concomitant treatment with corticosteroids may be suspected to exert some influence on the NLR, the exact determinants of the NLR remain to be fully elucidated.

## 5. Conclusions

Elevated levels of the NLR, as well as CRP, are significantly associated with prolonged myocardial T1 times and the expansion of the ECV. However, only a rather modest variation observed in both target parameters may be explained by sub-clinical systemic inflammation assessed using either the NLR or the CRP.

## Figures and Tables

**Table 1:** The patient cohorts baseline characteristics stratified by tertiles of the neutrophil–lymphocyte ratio.

Variable	Total Cohort (*n* = 1152)	NLR Tertile 1 ≤2.5 (*n* = 396)	NLR Tertile 2 >2.5 and <4.0 (*n* = 372)	NLR Tertile 3 ≥4.0 (*n* = 384)	*p*-Value
Demographics and Clinical Parameters					
Age, median (IQR)	72.4 (57.4–79.7)	67.8 (48.9–78.2)	72.6 (58.4–79.4)	75.3 (65.7–81.2)	<0.001
Male sex, n (%)	612 (53.1%)	199 (50.3%)	193 (51.9%)	220 (57.3%)	0.121
BMI, median (IQR)	26.4 (23.4–29.8)	26.6 (23.8–29.8)	26.7 (23.4–30.3)	26.1 (23.0–29.2)	0.262
Comorbidities and medical history					
Atrial fibrillation, n (%)	289 (28.0%)	71 (20.6%)	103 (30.0%)	115 (33.3%)	<0.001
Arterial hypertension, n (%)	651 (56.5%)	220 (55.6%)	201 (54.0%)	230 (59.9%)	0.238
COPD, n (%)	77 (6.7%)	15 (3.8%)	19 (5.1%)	43 (11.2%)	<0.001
Hyperlipidemia, n (%)	384 (33.3%)	131 (33.1%)	123 (33.1%)	130 (33.9%)	0.965
Hypercholesterinemia, n (%)	12 (1.0%)	6 (1.5%)	3 (0.8%)	3 (0.8%)	0.519
Diabetes mellitus, n (%)	205 (17.8%)	52 (13.1%)	66 (17.7%)	87 (22.7%)	0.002
Chronic kidney disease, n (%)	397 (42.5%)	95 (30.5%)	144 (44.0%)	167 (52.7%)	<0.001
Myocardial infarction, n (%)	333 (32.3%)	86 (25.0%)	115 (33.5%)	132 (38.3%)	<0.001
Significant CAD, n (%)	137 (32.6%)	53 (31.4%)	44 (33.1%)	40 (33.9%)	0.895
Previous PCI, n (%)	60 (14.3%)	24 (14.2%)	21 (15.9%)	15 (12.7%)	0.770
Previous CABG, n (%)	28 (6.6%)	7 (4.1%)	10 (7.5%)	11 (9.2%)	0.200
Smoking, n (%)	53 (14.6%)	23 (16.4%)	18 (15.7%)	12 (11.2%)	0.483
Laboratory Assessment and Markers of Inflammation					
NLR, median (IQR)	3.11 (2.15–4.67)	1.88 (1.50–2.16)	3.12 (2.80–3.53)	5.31 (4.67–6.90)	<0.001
CRP, mg/dL, median (IQR)	0.28 (0.11–0.73)	0.19 (0.08–0.43)	0.32 (0.11–0.78)	0.41 (0.16–1.25)	<0.001
Neutrophils, G/L, median (IQR)	4.60 (3.60–5.90)	3.55 (2.90–4.40)	4.75 (3.90–5.80)	5.95 (4.80–7.50)	<0.001
Lymphocytes, G/L, median (IQR)	1.50 (1.10–1.92)	2.00 (1.60–2.32)	1.50 (1.30–1.83)	1.00 (0.80–1.30)	<0.001
Thrombocytes, G/L, median (IQR)	222 (185–268)	216 (180–259)	223 (189–272)	229 (190–281)	0.017
Interleukin 6, pg/mL, median (IQR)	10.6 (3.44–42.8)	3.73 (1.87–20.3)	11.1 (5.08–36.2)	20.7 (6.64–79.4)	0.132
LDL, mg/dL, median (IQR)	84.5 (61.6–113)	91.4 (67.4–117)	80.3 (57.9–109)	82.6 (57.4–116)	0.051
NT-proBNP, pg/mL, median (IQR)	939 (288–2469)	610 (143–1695)	896 (290–2086)	1453(578–3911)	<0.001
Creatinine, mg/dL, median (IQR)	0.99 (0.82–1.29)	0.92 (0.79–1.13)	1.00 (0.83–1.29)	1.08 (0.88–1.48)	<0.001
eGFR, mL/min/1.73 m^2^, median (IQR)	65.7 (47.0–86.2)	78.2 (56.6–92.1)	63.7 (47.2–84.8)	57.9 (41.3–77.0)	<0.001

BMI, body-mass index; CABG, coronary artery bypass graft; CAD, coronary artery disease; COPD, chronic obstructive pulmonary disease; CRP, C-reactive protein; eGFR, estimated glomerular filtration rate; IQR, interquartile range; LDL, low-density lipoprotein; NLR, neutrophil–lymphocyte ratio; NT-proBNP, N-terminal prohormone of B-type natriuretic peptide; PCI, percutaneous coronary intervention. For normally distributed continuous variables, mean and standard deviation are reported and the *t*-test was used for comparison. For non-normally distributed variables, median and interquartile range are reported and the Kruskal–Wallis test was utilized.

**Table 2:** The cardiac magnetic resonance imaging characteristics at baseline stratified by tertiles of the neutrophil–lymphocyte ratio.

Variable	Total Cohort (*n* = 1152)	NLR Tertile 1 ≤2.5 (*n* = 396)	NLR Tertile 2 >2.5 & <4.0 (*n* = 372)	NLR Tertile 3 ≥4.0 (*n* = 384)	*p*-Value
Left Ventricle					
LVEDV, mL, median (IQR)	152 (118–197)	146 (117–197)	152 (120–190)	154 (118–201)	0.531
LVEDVi, mL/m^2^, median (IQR)	80.2 (64.2–101)	77.5 (62.6–101)	78.9 (64.2–98.4)	85.3 (67.0–104)	0.159
LVEF, %, median (IQR)	59.0 (50.0–67.0)	61.0 (51.0–68.0)	60.0 (52.0–68.0)	57.0 (46.0–65.0)	<0.001
LVCO, mL/min, median (IQR)	5.40 (4.20–6.70)	5.00 (4.50–6.40)	5.90 (4.75–6.80)	5.00 (4.00–7.00)	0.203
LVCOi, ml/min/m^2^, median (IQR)	2.87 (2.39–3.44)	2.88 (2.44–3.34)	2.92 (2.46–3.47)	2.85 (2.25–3.53)	0.413
IVS, mm, median (IQR)	11.0 (10.0–14.0)	11.0 (10.0–13.0)	12.0 (10.0–14.0)	12.0 (10.0–14.0)	0.004
LV mass, g, median (IQR)	133 (104–169)	128 (100–165)	131 (107–165)	137 (108–176)	0.175
LV mass index, g/m^2^, median (IQR)	71.4 (57.2–89.8)	71.4 (56.0–86.9)	70.1 (56.8–85.1)	72.7 (58.0–97.9)	0.129
Right Ventricle					
RVEDV, mL, median (IQR)	146 (118–187)	140 (114–177)	150 (119–195)	153 (125–193)	0.021
RVEDVi, mL, median (IQR)	77.8 (64.8–95.7)	75.6 (62.9–89.5)	77.4 (65.4–95.9)	80.9 (66.8–100)	0.006
RVEF, %, median (IQR)	54.0 (46.0–60.0)	56.0 (50.0–61.0)	54.0 (47.0–61.0)	51.0 (44.0–58.0)	<0.001
RVCO, mL/min, median (IQR)	5.00 (4.00–6.18)	5.00 (4.00–6.00)	5.00 (4.00–6.40)	5.00 (4.00–6.20)	0.182
RVCOi, mL/min, median (IQR)	2.66 (2.19–3.24)	2.63 (2.24–3.19)	2.71 (2.25–3.39)	2.67 (2.12–3.27)	0.381
Atria					
LAV, mL, median (IQR)	37.8 (33.5–41.9)	36.9 (32.9–41.9)	37.8 (33.7–41.9)	38.5 (34.3–42.5)	0.059
LAVi, mL/m^2^, median (IQR)	19.9 (17.5–22.9)	19.6 (17.2–22.7)	19.4 (17.1–22.8)	20.4 (17.8–23.1)	0.027
RAV, mL, median (IQR)	34.3 (30.8–38.5)	34.2 (30.5–38.5)	34.1 (31.0–38.7)	34.9 (31.2–38.4)	0.766
RAVi, mL, median (IQR)	17.9 (16.0–20.3)	17.7 (15.9–20.0)	18.0 (15.9–20.9)	18.0 (16.5–20.2)	0.930
Tissue Characteristics					
Myocardial native T1, ms, median (IQR)	1017 (989–1049)	1010 (984–1038)	1015 (986–1049)	1030 (1001–1059)	<0.001
Extracellular volume fraction, %, median (IQR)	27.0 (24.6–29.7)	26.3 (24.2–28.6)	26.7 (24.6–29.2)	28.1 (25.5–31.4)	<0.001

LV, left ventricular; RV right ventricular; -i, indexed to body surface area; CO, cardiac output; EDV, end-diastolic volume; EF, ejection fraction; IQR, interquartile range; IVS, interventricular septum; LAV, left atrial volume; NLR, neutrophil–lymphocyte ratio; RAV, right atrial volume. For normally distributed continuous variables, mean and standard deviation are reported and the *t*-test was used for comparison. For non-normally distributed variables, median and interquartile range are reported and the Kruskal–Wallis test was utilized.

**Table 3:** Univariable linear regression model for myocardial native T1 times.

Variable	β	95%-CI	R^2^	*p*-Value
NLR	2.42	1.36–3.49	0.017	<0.001
NLR Z-score	6.38	3.58–9.17	0.017	<0.001
C-reactive protein	6.77	3.79–9.76	0.017	<0.001
C-reactive protein Z-score	6.39	3.57–9.21	0.017	<0.001
Age	0.432	0.266–0.597	0.023	<0.001
Male sex	−5.52	−11.2–0.148	0.003	0.056
Body-mass index	−0.519	−0.961–−0.077	0.006	0.021
Atrial fibrillation	7.81	1.23–14.4	0.005	0.020
COPD	15.9	4.74–27.1	0.007	0.005
Hyperlipidemia	−7.93	−13.9–−1.96	0.006	0.009
Hypercholesterinemia	17.3	−10.3–44.9	0.001	0.218
Diabetes mellitus	4.37	−3.00–11.7	0.001	0.245
Chronic kidney disease	11.8	5.48–18.2	0.014	<0.001
Leukocyte count	−0.867	−2.28–0.550	0.002	0.230
NT-proBNP *	0.234	0.180–0.287	0.075	<0.001
eGFR	−0.293	−0.407–−0.179	0.027	<0.001

CI, confidence interval; COPD, chronic obstructive pulmonary disease; eGFR, estimated glomerular filtration rate; NT-proBNP, N-terminal prohormone of b-type natriuretic; * per 100 pg/mL.

**Table 4:** Univariable linear regression model for myocardial extracellular volume fraction.

Variable	β	95%-CI	R^2^	*p*-Value
NLR	0.297	0.179–0.416	0.023	<0.001
NLR Z-score	0.782	0.471–1.093	0.023	<0.001
C-reactive protein	0.914	0.569–1.26	0.026	<0.001
C-reactive protein Z-score	0.862	0.536–1.19	0.026	<0.001
Age	0.048	0.029–0.067	0.023	<0.001
Male sex	−0.152	−0.798–0.494	<0.001	0.645
Body-mass index	−0.060	−0.108–−0.013	0.008	0.013
Atrial fibrillation	2.13	1.44–2.82	0.039	<0.001
COPD	2.23	0.977–3.48	0.012	0.001
Hyperlipidemia	−0.581	−1.26–0.094	0.003	0.092
Hypercholesterinemia	0.306	−2.81–3.43	<0.001	0.847
Diabetes mellitus	0.797	−0.031–1.63	0.004	0.059
Chronic kidney disease	1.60	0.918–2.28	0.025	<0.001
Leukocyte count	−0.143	−0.300–0.010	<0.004	0.070
NT-proBNP *	0.027	0.020–0.034	0.067	<0.001
eGFR	−0.039	−0.052–−0.026	0.039	<0.001

CI, confidence interval; COPD, chronic obstructive pulmonary disease; eGFR, estimated glomerular filtration rate; NT-proBNP, N-terminal prohormone of b-type natriuretic; * per 100 pg/mL.

## Data Availability

Data is available upon reasonable request to the corresponding author.

## Abbreviations

The following abbreviations are used in this manuscript:

CI	confidence interval
CMR	cardiac magnetic resonance imaging
CRP	C-reactive protein
ECV	extracellular volume fraction
eGFR	estimated glomerular filtration rate
IQR	interquartile range
LDL	low-density lipoprotein
NLR	neutrophil–lymphocyte ratio
NT-proBNP	N-terminal prohormone of B-type natriuretic peptide
SD	standard deviation
